#  Efficacy of Mupirocin and Rifampin Used with Standard Treatment in the Management of Acne Vulgaris

**Published:** 2013

**Authors:** Farzin Khorvash, Fatemeh Abdi, Hessam H.Kashani, Farahnaz Fatemi Naeini, Fariborz Khorvash

**Affiliations:** a *Nosocomial Infection Research Center, Isfahan University of Medical Sciences, Isfahan, Iran.*; b *Department of Reproductive Health, Faculty of Nursing and Midwifery , Shahid Beheshti University of Medical Sciences, Tehran, Iran. *; c *Department of Physiology, Faculty of Medicine,University of Manitoba, Winnipeg, Manitoba, Canada. *; d *Depatment of Skin Diseases, Isfahan University of Medical Sciences, Isfahan, Iran. *; e *Department of Neurology, Faculty of Medicine, Isfahan University of Medical Sciences, Isfahan, Iran. *

**Keywords:** Acne vulgaris, Rifampin, Mupirocin, Doxycyclin, GAGS

## Abstract

The multiple etiologic factors involved in acne make the use of various medications necessary to treat the condition. This study aimed to determine the efficacy of mupirocin and rifampin used with standard treatment in the management of acne vulgaris. In a multicentre, randomized controlled, triple-blinded study, a total of 105 acne patients, with a clinical diagnosis of moderate to severe acne,were randomizedly divided into three groups (35 per group), for treatment of acne. The first group was treated with standard treatment alone, the second group received mupirocin plus standard treatment and the third group received rifampin plus standard treatment.There were three study visits according to Global Acne Grading System (GAGS): at baseline and weeks 6 and 12. The absolute changes of GAGS score from baseline to week 6 and 12 demonstrated a reduction in the mean score of GAGS in the three treatment groups (p < 0.001). Due to the difference between GAGS score at the baseline of study, the data were adjusted using the general linear model. The findings showed that all of the treatments significantly improved acne lesions. Nevertheless, none of the treatments was shown to be more effective than the others (p = 0.9). The three treatments were well tolerated, and no serious adverse events were reported. These findings provide evidence on the efficacy of combining mupirocin and rifampin with standard treatment in the management of acne vulgaris, although none of the treatments had superior efficacy compared with the others.

## Introduction

Acne vulgaris is the most common disorder of human skin that affects up to 80% of adolescents (1). Several studies suggest that the emotional impact of acne is comparable with disabling diseases, such as diabetes and epilepsy (2). Acne has a negative effect on the quality of life; although this can be improved with effective treatment (3). Acne has four main pathogenetic mechanism:increased sebum productions, follicular hyperkeratinization, Propionibacterium acne (P. acne) colonization, and the products of inflammation (4). 

Many medications are available for the management of acne. Antibiotic therapy has been integral to the treatment of acne for many years. The widespread and long-term use of antibiotics has unfortunately led to the emergence of resistant bacteria (5). Combination therapy with a topical retinoid and an antibiotic is recognized as an effective method for the management of acne vulgaris. The combination of adapalene with oral or topical antibiotics has been shown to release a faster response than an antibiotic alone (6). Clinical studies have shown some success with a combition of doxycycline and adapalene (7). 

In the other hand, there is significant *in-vitro *evidence suggesting a possible pathogenetic role for *Staphylococcus aureus *(*S. aureus*) in acne vulgaris (8). Recent advances in the pathogenesis of acne have led to the development of new therapeutic goals (9). Long-term therapy with oral antibiotic is not only a threat to resistant of P. acne, but also to coagulase negative staphylococci on the skin, *S. aureus *in the nares, and streptococci in the oral cavity (4).

In the analysis of the bacterial flora of acne lesions, P. acnes and Staphylococcus epidermidis were found to be sensitive to the antibiotics used in the treatment of acne. The highest antibacterial effect against both these species was demonstrated when using rifampin and tetracycline (10). 

Considering the development of resistance in microorganisms causing acne to antibiotics,we designed a randomized controlled trial to investigate whether combining mupirocin and rifampin with standard treatment had faster effects than the standard treatment in the management of acne. Standard treatment consisted of doxycycline 100 mg, clindamycin 1% and adapalene 0.1%, as the initial therapy for acne vulgaris.

## Experimental

This was a multicentre, randomized controlled, triple-blinded and parallel comparison study. The subjects referred to the dermatology clinic , with a clinical diagnosis of moderate to severe acne, were enrolled in this study. Patients were examined for the presence of acne by a dermatologist, according to to Global Acne Grading System (GAGS). A score of 1–18 was considered as mild acne, 19–30 as moderate, 31–38 as severe, and above 39 as very severe (11). Patients with chronic diseases, acne fulminans, GAGS scores greater than 39 or lower than 19, pregnant and nursing women, presence or history of active malignancy, under immunosuppressive treatment, and with mental incapacity, were excluded. Specified appropriate washout periods were required for patients taking certain topical and systemic treatments. For topical agents the washout period was 4 weeks (for corticosteroids, retinoids and other acne treatments); and 8 weeks (for chemical peeling, laser and light-based therapies). For systemic drugs, the washout periods were 12 weeks for corticosteroids and other acne treatments. Ethical approval was taken from the ethical committee of Isfahan University of Medical Sciences. Written informed consent was obtained from the participants.

Patients were randomly allocated to the three treatment groups, using a random numbers table. The first group was treated with standard treatment alone, the second group with mupirocin plus standard treatment and the third group with rifampin plus the standard treatment. Patients were enrolled at baseline and treated daily for 12- weeks. Standard treatment consisted of a combination of doxycycline 100 mg (twice daily) and clindamycin solution 1% (twice daily) and adapalene gel 0.1% (once daily). The second group received intra-nasal mupirocin ointment 1% (twice daily) for the first ten days of the 12 weeks standard therapy. The third group received oral rifampin 300 mg (twice daily) for the first ten days of the 12 weeks standard therapy. There were three study visits: at baseline and weeks 6 and 12. At the beginning of the study there were 210 participants (70 per group), but half of them were excluded out of the study due to their loss for follow up, interfering medication and a lack of compliance to the visit schedule. Finally, a total of 105 patients (35 per group), completed the study. 

The acne lesions were assessed by a physician, who was blinded to the patients’ treatment. GAGS was counted during each visit and efficacy of each treatment was investigated by comparing the grade of acne lesions at each visit, with the baseline value. At each visit patients were clinically assessed with a checklist for adverse events attributable to every treatment.The coding method was used for blinding. Patient, investigator and the physician performing examinations during the follow up were not informed about about the treatment allocation for each patient, which leads to this unique triple blinded study design. 

*Statistics*

Data gathered were analyzed using the one-way ANOVA, Chi-Square and Mann-Whitney tests and through Statistical Package for the Social Sciences (SPSS) software version 15.0, by considering p-values of < 0.05 as statistically significant. 

## Results

The 105 patients who fulfilled the inclusion criteria, continued to participate in this study. The studied group consisted of 84 females (80%) and 21 males (20%), with a mean age of 23.8 ± 6.1 years. In the present study, their ages ranged from 16 to 35 years and the difference between groups in term of the demographic characteristics was statistically insignificant. 

**Figure 1:**
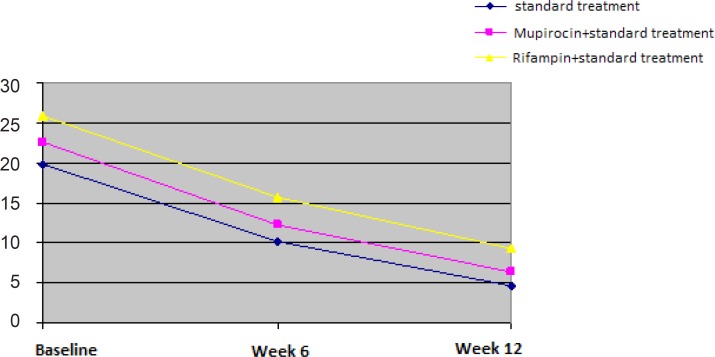
changes in the GAGS scores during the study visit

Table 1 provides the mean score of GAGS at baseline and weeks 6 and 12, after the initiation of treatment in the three groups. The absolute changes from baseline to week 6 on the basis of GAGS score were 10.2 ± 6 with the standard treatment, 12.2 ± 7.3 with mupirocin plus standard treatment and 15.7 ± 6.1 with rifampin plus the standard treatment. At week 12, the mean scores of GAGS decreased from 19.8 ± 7.7 to 4.6 ± 5.9 in the first group, from 22.6 ± 6 to 6.3 ± 5.5 in the second group, and from 25.8 ±5.2 to 9.3 ±5.5 in the third group. Considering the changes in the mean score of GAGS at weeks 6 and 12, a reduction in the mean score of GAGS in the three treatments (p < 0.001) (Figure 1) was observed. This is while the two groups of mupirocin and rifampin were not significantly different regarding to the decrease in GAGS scores (p > 0.05), (Table 1). Due to difference between GAGS score at the baseline of the study, the data was adjusted using the general linear model, for the purpose of comparison. The findings of this study showed that all the treatments significantly improved acne lesions. Nevertheless, none of the treatments was shown to be more effective than the others (p = 0.9). 

**Table 1:** Comparison of the changes in the GAGS scores in the three test groups

**group**	**Standard treatment **	**Mupirocin+ standard treatment **	**Rifampin + standard treatment **	**p-value**
**visit**				
Baseline	19.8 ± 7.7	22.6 ± 6	25.8 ± 5.2	< 0.001
Week 6	10.2 ± 6	12.2 ±7.3	15.7 ± 6.1	< 0.001
Week 12	4.6 ± 5.9	6.3 ± 5.5	9.3 ± 5.5	< 0.001

Moreover, the findings showed that the three treatments were well tolerated, and no serious adverse events were reported. In the standard therapy group, five patients and in the mupirocin group used with the standard therapy three patients had gastrointestinal irritation. In rifampin plus the standard therapy goupe, two patients had photosensitivity (p = 0.44).

## Discussion

Acne is a multifactorial disease of as yet incompletely elucidated etiology and pathogenesis (12). The management of acne remains a global problem and treatment options are far from ideal (13). In the current study, all of the administered treatments indicated a meaningful reduction in acne severity (GAGS), among all three groups. Most of the published studies have reported that systemic antibiotics have been found test to be useful in managing moderate to severe acne (14). Thiboutot *et al*. reported that combining adapalene with an oral antibiotic provides a superior advantage over the use of antibiotic alone and should be considered at the onset of treatment (6). However ,increased resistance to systemic and topical antibiotics have been reported in the performed studies in America (15), Italy, Greece (16), Japan and Australi ( 17). 

The multiple etiologic factors involved in acne, make the use of various medications necessary to treat the condition (18). Combination therapy is the standard of care in the treatment of acne. It is essential to treat as many aspects of acne pathogenesis (19). Combining agents that target the different etiological factors of acne can help to increase the efficacy and response time (7). In a recent study, 25% of acne patients had *S.aureus *colonization solely in their nose; and 19% had *S. aureus *in both their nose and their throat ( 20). Effect of mupirocin on *S.aureus *has been established and it can eradicate the *S.aureus *in nasal carriages (21). Intra-nasal mupirocin is well tolerated and has an obvious effect in eradicating of *S.aureus *in the nasal carriage, as well as Rifampin has a similar effect on removing staphylococcus from the nose (22).

Our final findings indicated that a combination of mupirocin and rifampin alongside the standard treatment had no superior efficacy, compared with athe others. In this respect, based on our literature review it seems that the of mupirocin or rifampin in acne treatment has been considered for the first time in our study. Because of the few trials available, it is impossible to compare our results with the other studies. In fact, this hypothesis should be investigated by conducting future investigations.

Due to the growing concerns of rising antibiotic resistance, and the lack of safe and effective agents (9), treatment options and follow up procedures in acne should be carefully determined to reduce the risk of destruction of the microbial flora (23). The choice of antibacterial should take into account the severity of the acne, cost effectiveness, risk-benefit ratios, and the potential for the development of resistance (14).

To the best of our knowledge, there has been no similar study on the combination therapy with mupirocin and rifampin in the management of acne. It is noteworthy to mention that the most important issues of the present study were the design and to implemention of an accurate methodology and paying respect to the principles of blinding. Limitations of the present study were high rate of patient loss and very little published evidence.

In conclusion, the use of standard treatment, either in combination with mupirocin and Rifampin or alone for acne management seems to be effective without any important side effects, and no superiority was observed between the combination and solo therapy.

## Notes

Authors have no conflict of interests in the study.
